# Smoking in public places in six European countries: Findings from the EUREST-PLUS ITC Europe Survey

**DOI:** 10.18332/tid/104673

**Published:** 2019-03-28

**Authors:** Marcela Fu, Yolanda Castellano, Olena Tigova, Ute Mons, Thomas Agar, Christina N. Kyriakos, Antigona C. Trofor, Anne C. K. Quah, Geoffrey T. Fong, Krzysztof Przewoźniak, Witold A. Zatoński, Tibor Demjén, Yannis Tountas, Constantine I. Vardavas, Esteve Fernández, on behalf of the EUREST-PLUS consortium

**Affiliations:** 1 Catalan Institute of Oncology (ICO), L’Hospitalet de Llobregat, Spain; 2 Bellvitge Biomedical Research Institute (IDIBELL), L’Hospitalet de Llobregat, Spain; 3 University of Barcelona (UB), Barcelona, Spain; 4 Cancer Prevention Unit and WHO Collaborating Centre for Tobacco Control, German Cancer Research Center (DKFZ), Heidelberg, Germany; 5 University of Waterloo (UW), Waterloo, Canada; 6 European Network on Smoking and Tobacco Prevention (ENSP), Brussels, Belgium; 7 University of Crete (UoC), Heraklion, Greece; 8 University of Medicine and Pharmacy ‘Grigore T. Popa’ Iasi (UMF Iasi), Iasi, Romania; 9 Aer Pur Romania (APR), Bucharest, Romania; 10 Ontario Institute for Cancer Research (OICR), Toronto, Canada; 11 Health Promotion Foundation (HPF), Warsaw, Poland; 12 Maria Skłodowska-Curie Institute - Oncology Center (MSCI), Warsaw, Poland; 13 European Observatory of Health Inequalities, President Stanisław Wojciechowski State University of Applied Sciences (PSWZ), Kalisz, Poland; 14 Smoking or Health Hungarian Foundation (SHHF), Budapest, Hungary; 15 National and Kapodistrian University of Athens (UoA), Athens, Greece

**Keywords:** smoking, smoke-free legislation, workplaces, leisure facilities, hospitality venues

## Abstract

**INTRODUCTION:**

Surveillance of tobacco consumption in public places is an important measure to evaluate the impact of tobacco control interventions over time. The objective of this study was to estimate the prevalence of smoking as seen by smokers and their smoking behaviour in public places, in six European countries.

**METHODS:**

We used baseline data of the International Tobacco Control Six European countries (ITC 6E) Survey, part of the EUREST-PLUS Project, conducted in 2016 in national representative samples of about 1000 adult smokers aged 18 years and older in Germany, Greece, Hungary, Poland, Romania and Spain. For each setting (workplaces, restaurants, bars/pubs and discos) participants were asked whether they had seen someone smoking during their last visit there and whether they too had smoked there. We report the overall and by-country weighted prevalence of seeing someone smoking and the smokers’ own smoking behaviour at each setting. We also assess the relationship between seeing someone smoking and smoking themselves at these settings.

**RESULTS:**

The prevalence of smoking as seen by smokers was 18.8% at workplaces, with high variability among countries (from 4.7% in Hungary to 40.8% in Greece). Among smokers visiting leisure facilities in the last year, during their last visit 22.7% had seen someone smoking inside restaurants and 12.2% had smoked themselves there, while for bars/pubs the corresponding prevalences were 33.9% and 20.4%, and inside discos 44.8% and 34.8%.

**CONCLUSIONS:**

Smoking is still prevalent at leisure facilities, particularly at discos in Europe, with high variability among countries. More extensive awareness campaigns and stricter enforcement are needed to increase the compliance of smoke-free regulations, especially in leisure facilities.

## INTRODUCTION

Tobacco smoking is the main individual preventable cause of premature morbidity and mortality worldwide, being a risk factor for six of the eight leading causes of death in the world^1^. The World Health Organization Framework Convention on Tobacco Control (WHO FCTC) provides some guidelines for tobacco control at the national level, known as the MPOWER policy package. MPOWER includes specific measures to implement effective interventions to reduce tobacco demand^1^. Following the MPOWER guidelines, many countries have implemented extensive smoke-free regulations since 2004, with important reductions in the exposure to secondhand tobacco smoke in public places and improved health outcomes, although more discrete results in the smoking prevalence and tobacco consumption have been observed^2,3^.

Literature reviews of studies about the effects of smoke-free policies on the population’s health conclude that long-term assessment provides more robust results for the effects of such policies^2-4^. One of the measures included in the MPOWER policy package is the surveillance of tobacco consumption and the subsequent exposure to secondhand smoke in the population. Several surveys have been conducted at the population level after the implementation of national legislations^5^, but the perceptions of smokers and whether they smoke or not in public places have not been systematically assessed in representative samples of smokers. For this reason, this study aims at describing the prevalence of smoking as seen by smokers and their own smoking behaviour in selected public places in six European countries with different smoke-free legislations.

## METHODS

### Study design

We used the baseline information of the EUREST-PLUS Project (https://eurestplus.eu/), aiming at assessing the impact of the Tobacco Products Directive at the European level. We set up a representative sample of 6011 smokers from Germany, Greece, Hungary, Poland, Romania and Spain (about 1000 smokers in each country), within the International Tobacco Control Policy Evaluation (ITC) Project (www.itcproject.org/). This cohort survey (ITC 6E Survey) follows the methods of the ITC Project, explained in detail elsewhere^6,7^. Briefly, samples of adult (≥18 years old) current smokers (having smoked >100 cigarettes in their lifetimes and having smoked at least once in the past 30 days) were recruited by probability sampling methods, being representative of all geographical regions in each country. Households were selected using a random walk procedure and considered to be eligible if it included at least one eligible smoker. Where available, both one male and one female smoker were selected from each household using the last birthday method^8^. The survey was conducted between June and September 2016. After informed consent was provided, a computer-assisted personal interview (CAPI) was conducted. The study protocol was approved by an ethics committee in each participating country.

### Measures

#### Smoking at work

Among smokers who were employed outside the home at the survey time the following question was asked: ‘In the last 30 days, have people smoked in indoor areas where you work?’, with ‘yes’ and ‘no’ being the possible answers.

#### Seeing someone smoking at leisure facilities

For each of the three types of leisure facilities assessed, smokers were first asked: ‘In the last 12 months, how often have you visited [a restaurant/a drinking establishment such as a pub or bar/a nightclub or disco] where you live?’, with answer options ‘more than once a week, ‘about once a week’, ‘about once or twice a month’, ‘less than once a month’ and ‘never’. Smokers who answered any of the options except ‘never’ were included in this specific analysis describing smoking at each setting. To ascertain information about whether they had seen someone smoking at these settings in the last 12 months, they were asked: ‘The last time you did so, were people smoking inside [the restaurant/ the pub or bar/the nightclub or disco]?’. The possible answers were ‘yes’ and ‘no’.

#### Smoking themselves at leisure facilities

Additionally, information about whether they had smoked at the same settings was ascertained with the question: ‘Did you smoke at all at [the restaurant/the pub or bar/the nightclub or disco], including both inside or outside, during your last visit?’. The possible answers were ‘yes’ and ‘no’. For those answering ‘yes’, the following question was asked: ‘Did you smoke inside [the restaurant/ the pub or bar/the nightclub or disco], outside or both?’, possible answers were ‘inside only’, ‘outside only’ and ‘both inside and outside’. The first and third possible answers were considered for the analysis of the information about their own smoking behaviour inside leisure facilities.

### Analysis

We describe the overall and by-country prevalence rates (and 95% confidence intervals, CI) of seeing someone smoking inside all the studied settings (workplaces, restaurants, bars/pubs and discos). We also describe the respondents’ smoking behaviour inside all leisure facilities. Finally, we assess the relationship between the prevalence of seeing someone smoking and smoking themselves inside all leisure facilities. All the analyses incorporated the weights derived from the complex sampling design. We used Stata v.13 for all analyses.

## RESULTS

### Seeing someone smoking at work and leisure facilities

Among smokers who were employed outside the home (n=3524), 18.8% (95% CI: 17.2-20.3%) had seen someone smoking inside their workplaces in the last 30 days. The lowest prevalence was reported in Hungary (4.7%; 95% CI: 2.7-6.6%) and the highest in Greece (40.8%; 95% CI: 35.8-45.8%) (Table 1). Among those smokers who reported to have visited each leisure facility studied in the last 12 months, during their last visit on average 22.7% had seen someone smoking inside restaurants (ranging from 3.4% in Spain to 71.7% in Greece), 33.9% inside bars/pubs (from 5.5% in Hungary to 87.8% in Greece) and 44.8% inside discos (from 9.3% in Hungary to 89.4% in Greece) the last time they visited these settings (Table 1).

**Table 1:** Prevalence (%)^a^ of seeing someone smoking at workplaces in the last 30 days and during the last visit to some leisure facilities within the last 12 months in six European countries, 2016

	*Workplaces*			*Restaurants*			*Bars/Pubs*			*Discos*		
	*n*	*%*	*95% CI*	*n*	*%*	*95% CI*	*n*	*%*	*95% CI*	*n*	*%*	*95% CI*
Overall	3524	18.8	17.2-20.3	3769	22.7	20.9-24.4	3905	33.9	31.7-36.1	1737	44.8	41.5-47.7
Germany	660	17.2	12.9-21.5	875	12.6	9.4-15.6	728	38.4	30.7-46.1	286	53.0	45.6-60.4
Greece	577	40.8	35.8-45.8	820	71.7	65.1-78.4	872	87.8	81.9-93.6	482	89.4	82.8-96.0
Hungary	668	4.7	2.7-6.6	386	7.7	4.2-11.1	417	5.5	2.1-8.9	149	9.3	4.3-14.4
Poland	508	23.1	18.5-27.6	374	8.4	6.0-11.5	440	25.4	19.8-30.9	219	27.0	19.3-33.7
Romania	563	20.1	16.4-23.8	512	12.1	9.1-14.9	561	15.1	11.4-18.8	179	19.3	13.2-25.5
Spain	548	10.2	7.3-13.1	802	3.4	2.1-4.6	887	8.0	6.0-10.0	422	21.4	16.4-26.5

a Weighted prevalences. CI: confidence interval.

### Respondents smoking themselves at leisure facilities

On average, 12.7% of smokers had smoked inside restaurants in the last 12 months (ranging from 0.6% in Hungary to 46.4% in Greece), 21.3% inside pubs/ bars (from 1.8% in Hungary to 56.3% in Greece), and 35.2% inside discos (from 2.1% in Hungary to 81.1% in Greece) (Table 2).

**Table 2:** Prevalence (%)^a^ of smokers who smoked indoors during their last visit to some leisure facilities within the last 12 months in six European countries, 2016

	*Restaurants*			*Bars/Pubs*			*Discos*		
	*n*	*%*	*95% CI*	*n*	*%*	*95% CI*	*n*	*%*	*95% CI*
Overall	3777	12.7	11.3-13.9	3915	21.3	19.5-23.0	1734	35.2	32.3-38.0
Germany	874	4.0	1.6-6.2	730	27.7	21.2-33.9	287	37.3	28.8-45.4
Greece	823	46.6	41.4-51.5	873	56.4	51.6-61.0	482	81.1	76.9-85.3
Hungary	391	0.6	0.0-1.5	418	1.8	0.0-3.8	148	2.1	0.0-4.7
Poland	374	2.6	1.2-3.9	443	13.7	9.6-17.5	217	16.1	9.3-22.7
Romania	512	6.9	4.5-9.4	563	8.7	6.0-11.4	179	13.8	7.9-19.8
Spain	803	1.1	0.4-1.8	888	3.3	2.2-4.5	421	12.4	8.5-16.4

a Weighted prevalences. CI: confidence interval.

### Relationship between seeing someone smoking and smoking themselves at leisure facilities

Table 3 shows the relationship between having seen someone smoking and having smoked themselves at restaurants, pubs/bars and discos. About 23% of smokers on average had seen someone smoking during their last visit to a restaurant, of which 12.2% had smoked themselves at these venues and 10.5% had not. By country, these differences were more noticeable. In Germany, 3.6% had seen someone smoking inside restaurants and had smoked themselves, while 9.0% had seen someone smoking but they had not smoked themselves. Similarly, these prevalences were 45.9% and 25.8% in Greece, 0.6% and 7.1% in Hungary, and 2.0% and 6.4% in Poland, respectively. In contrast, 0.5% of all respondents had not seen someone smoking inside restaurants but had smoked themselves inside these facilities (Table 3).

**Table 3:** Relationship between seeing someone smoking and smoking themselves indoors during the last visit to some leisure facilities within the last 12 months in six European countries, 2016

*Smokers smoking at these settings*	*Seeing someone smoking at restaurants^a^*						*Seeing someone smoking at pubs/ bars^a^*						*Seeing someone smoking at discos^a^*					
	*Yes*			*No*			*Yes*			*No*			*Yes*			*No*		
	*n*	*%*	*95% CI*	*n*	*%*	*95% CI*	*n*	*%*	*95% CI*	*n*	*%*	*95% CI*	*n*	*%*	*95% CI*	*n*	*%*	*95% CI*
**Overall**																		
Yes	471	12.2	10.8-13.5	18	0.5	0.2-0.8	819	20.4	18.8-22.1	27	0.9	0.5-1.2	627	34.8	32.0-37.6	9	0.4	0.2-0.7
No	387	10.5	8.8-12.2	2884	76.8	75.1-78.6	539	13.5	11.7-15.3	2516	65.2	63.0-67.4	180	10.0	8.1-11.8	916	54.8	51.7-57.9
**Germany**																		
Yes	27	3.6	1.3-5.8	3	0.4	0.0-0.8	186	26.9	20.7-33.0	6	0.8	0.1-1.5	99	36.1	27.7-44.4	3	1.2	0.0-2.6
No	72	9.0	6.7-11.3	770	87.0	83.9-90.1	81	11.5	8.0-15.1	455	60.8	53.0-68.7	46	16.9	12.1-21.8	138	45.8	38.4-53.2
**Greece**																		
Yes	395	45.9	40.9-51.0	4	0.7	0.0-1.3	509	55.7	51.2-60.2	3	0.7	0.0-1.6	420	81.1	76.9-85.3	0	-	-
No	210	25.8	18.8-32.8	211	27.6	21.0-34.2	283	32.1	25.4-38.8	76	11.5	6.0-17.1	38	8.3	3.5-13.1	24	10.6	4.0-17.2
**Hungary**																		
Yes	3	0.6	0.0-1.5	0	-	-	6	1.4	0.0-3.2	2	0.4	0.0-1.2	3	1.7	0.0-4.2	1	0.4	0.0-1.1
No	22	7.1	3.9-10.3	361	92.3	88.9-95.8	21	4.1	1.7-6.4	386	94.1	90.5-97.7	11	7.6	3.2-12.1	133	90.3	85.1-95.5
**Poland**																		
Yes	7	2.0	0.9-3.1	3	0.6	0.0-1.2	50	12.2	8.3-16.0	6	1.5	0.4-2.6	30	15.7	9.1-22.3	1	0.4	0.0-1.0
No	28	6.4	3.7-9.1	332	91.0	88.0-94.0	61	13.2	9.4-17.1	322	73.1	67.5-78.7	30	11.3	7.1-15.5	155	72.6	65.1-80.2
**Romania**																		
Yes	33	5.7	3.8-7.7	6	1.2	0.0-2.7	35	6.5	4.2-8.8	8	2.2	0.7-3.7	23	13.2	7.6-19.0	1	0.6	0.0-1.7
No	34	6.4	3.7-8.9	439	86.7	83.2-90.3	49	8.6	5.4-11.7	469	82.7	79.0-86.4	13	6.1	2.4-9.8	142	80.1	73.7-86.4
**Spain**																		
Yes	6	0.8	0.2-1.4	2	0.3	0.0-0.7	33	3.2	2.1-4.4	2	0.1	0.0-0.3	52	11.9	8.0-15.8	3	0.5	0.0-0.9
No	21	2.6	1.3-3.8	771	96.3	95.0-97.7	44	4.8	3.2-6.3	808	91.9	89.8-93.9	42	9.5	6.6-12.4	324	78.1	72.9-83.3

a Weighted prevalences. CI: confidence interval.

At pubs/bars, 33.9% of respondents on average had seen someone smoking indoors, of which 20.4% had smoked there themselves and 13.5% had not. Significant differences were observed between these groups in Germany (26.9% had seen someone smoking and had smoked themselves, while 11.5% had seen someone smoking but had not smoked themselves) and in Greece (55.7% had seen someone smoking and had smoked themselves, while 32.1% had seen someone smoking but had not smoked themselves). Conversely, 0.9% of all respondents had not seen someone smoking at these settings but reported smoking themselves there during their last visit to these facilities within the last 12 months (Table 3).

At discos, about 45% of smokers on average had seen someone smoking, of which 34.8% had smoked there themselves and 10.0% had not. Statistically significant differences were observed between these groups in Germany (36.1% had seen someone smoking and had smoked themselves, while 16.9% had seen someone smoking but had not smoked themselves) and in Greece (81.1% had seen someone smoking and had smoked themselves, while 8.3% had seen someone smoking but had not smoked themselves) (Table 3). Overall, 0.4% of smokers had not seen someone smoking at discos but declared smoking there themselves (Table 3).

## DISCUSSION

Our results indicate that smoking in some public places is still highly prevalent, with some heterogeneity among countries. This is the case for workplaces, where about 20% of respondents reported seeing someone smoking there in the last 30 days; while smoking was scarcely seen in Hungary, it was highly seen in Greece. This overall prevalence is, however, lower than that observed in the data from the Eurobarometer of 2014, in which exposure to tobacco smoke at work was assessed in 28 European countries (35% of smokers were passively exposed)^9^. Differences by country observed in our data can be explained by the particularities of national legislations (Table 4). For example, in Greece, smoking is totally banned in public and private working areas since 2003, but the enforcement has not been totally effective and some failure in compliance is still present. In Germany, three of 16 state (Land) governments have introduced comprehensive smoke-free legislation, but all other states have only partial legislation, allowing exceptions for designated smoking rooms and smoking bars. In the rest of the countries, smoking is totally banned in all enclosed workplaces with few exceptions^10^. In Romania, the comprehensive law banning smoking in all enclosed public places, enclosed workplaces and children playgrounds came into effect in March 2016, just before the survey was conducted; thus, these specific results may indicate the level of compliance with the comprehensive law just after its implementation. Further information to be collected in successive waves of the EUREST-PLUS cohort will allow a better assessment of the level of compliance with the comprehensive law in Romania.

**Table 4:** Smoke-free legislation in the six participant countries of the EUREST-PLUS ITC Europe survey, with an indication of a total (T) or partial (P) ban in selected public places and the year of implementation

	*Germany*	*Greece*	*Hungary*	*Poland*	*Romania*	*Spain*
Workplaces (indoors)	P (2007)^a^	T (2003)	T (2012)^d^	P (2010)^e^	T (2016)^f^	T (2006)
Restaurants (indoors)	P (2007-2008)^a^	T (2003)	T (2012)	P (2010)^e^	T (2016)	T (2011)
Restaurants (outdoors)	None	None	None	None	None	P (2011)^g^
Pubs/Bars (indoors)	P (2007-2008)^b^	P (2003)^c^	T (2012)	P (2010)^e^	T (2016)	T (2011)
Pubs/Bars (outdoors)	None	None	None	None	T (2016)	P (2011)^g^
Discos/Nightclubs (indoors)	P (2007-2008)^b^	P (2003)^c^	T (2012)	P (2010)^e^	T (2016)	T (2011)
Discos/Nightclubs (outdoors)	None	None	None	None	T (2016)	P (2011)^g^

a Smoke-free legislation is regulated at the regional level. In most states, separate, enclosed smoking rooms are allowed.

b Smaller establishments that do not serve food are exempted from the smoking ban altogether.

c Smoking is allowed in entertainment centres >300 m^2^ with live music and casinos.

d Smoking rooms are allowed under certain conditions in certain types of workplaces with increased risk of fire and/or explosion.

e Total smoking ban in enclosed public places. Enclosed, ventilated smoking rooms allowed in the hospitality sector and other workplaces.

f Smoking rooms are allowed in some enclosed public places.

g Smoking is forbidden in terraces with a roof or ceiling and more than two walls. Source: European Commission^10^.

At leisure facilities, on average more than 20% of respondents had seen someone smoking indoors, with huge differences by country and type of setting. Smoking indoors was less seen over all facilities in Hungary (<10%) and more often seen in Greece (around 70 to 90%). The overall prevalence of seeing someone smoking was lower at restaurants and higher at discos. Prevalence of smoking inside bars/pubs was consistent with another recent study conducted in eight European countries that also recorded to have seen smoking during bar visits^11^. Smoking at discos was highly prevalent, probably because smoking at these settings is more associated with tolerance and absence of formal rules, and because they are settings mainly frequented by young people. Besides, the phenomenon of ‘social smoking’ at discos may occur to a greater extent than at other leisure facilities among occasional smokers, those who smoke only when socialising. Another possible explanation of the high prevalence of smoking at discos may be related to the fact that in some cases these types of venues may have more space compared to restaurants and pubs; thus, smokers may perceive the spaciousness of these settings as permission to smoke. The prevalences observed in our survey are higher compared to those reported in the recent Eurobarometer Survey of 2017, in which 20% of respondents (smokers and non-smokers from 28 European countries) declared to have seen someone smoking inside a bar and 9% inside a restaurant^12^, although these differences should be interpreted with caution because of the specificities of the survey (using a general population sample, including 28 European countries, etc.).

Smoking behaviour by the respondents themselves was less prevalent than seeing someone else smoking at all the studied settings, probably indicating a potential information bias due to social desirability. Nevertheless, it follows the same pattern of seeing someone smoking, that is, lower prevalence at restaurants, and higher prevalence at pubs/bars and especially at discos. This pattern might also be indicating the degree at which formal and informal control systems can operate in each of these settings.

There is no clear pattern in the smokers’ behaviour at places where they had seen someone smoking. For example, a higher percentage of smokers in Greece who had seen someone smoking inside restaurants had themselves smoked there, but most smokers in Germany, Hungary and Poland who had seen someone smoking had not smoked themselves there. On the other hand, at pubs/bars and discos, higher prevalence of smokers in Germany and Greece who had seen someone smoking inside these venues had smoked themselves there compared to those who had seen someone smoking but did not smoke themselves at these settings. Again, this seems to indicate differences in compliance with smoking regulations according to the different types of settings. A study assessed the compliance with national comprehensive smoke-free laws in 41 countries in 2014 (six countries from Europe); its results indicate that the level of compliance with a national comprehensive smoke-free law is related to the depth of the enforcement infrastructure and effort of the local government in training enforcement officials/agents or directing their inspections^13^.

Many factors can be implied in the degree of the regulations’ compliance, including the population’s knowledge of the regulations or proper signage, among others^14^. Thus, some policy actions may be promoted, such as periodical information campaigns addressed to the population, visible no smoking signage, particularly in leisure facilities, and inspections, as well as training in smoking prevention and tobacco control addressed to key social actors. Such activities may help to create and maintain smoking denormalisation and thus promote a reduction of smoking in public places.

### Limitations

This study has some limitations. First, given the cross-sectional nature of the design, we are not able to draw causal relations but associations. Second, the use of a questionnaire may lead to potential information bias, especially when asking smokers about their own smoking behaviour in public places. Third, some responses are based on what the respondents are able to remember over a period of 12 months; thus, recall bias might affect the results. Nevertheless, this study allows comparison of patterns among countries at a time when these regulations are well established in Europe. Also, it provides a snapshot from the smokers’ point of view.

## CONCLUSIONS

Smoking in public places is still prevalent in Europe, particularly at discos, with high variability among countries. More extensive awareness campaigns and better enforcement are needed to maintain and increase the compliance of smoke-free regulations, especially at leisure facilities.
